# Health Care Professionals’ Perspectives on Conversational Mental Health Chatbots: Protocol for a Systematic Review

**DOI:** 10.2196/92780

**Published:** 2026-08-14

**Authors:** Besran Zara Malgir, Yi Jiao (Angelina) Tian, Emma Herger, Stephen R Milford, David Shaw, Bernice Simone Elger

**Affiliations:** 1 Institute for Biomedical Ethics, Faculty of Medicine, University of Basel, Bernoullistrasse 28, Basel, 4056, Switzerland, 41 612071784; 2 Utrecht University, Utrecht, The Netherlands; 3 Faculty of Theology, North-West University, Potchefstroom, South Africa; 4 Care and Public Health Research Institute, Maastricht University, Maastricht, Limburg, The Netherlands; 5 Center for Legal Medicine, University of Geneva, Geneva, Switzerland

**Keywords:** artificial intelligence, AI, chatbots, mental health, conversational artificial intelligence, conversational AI, health care professionals

## Abstract

**Background:**

Mental health disorders (MHDs) represent a growing global challenge and pose a significant risk to public health. Alongside developments in the field of large language models (LLMs), conversational mental health chatbots (CMHBs) have emerged and are increasingly being used by individuals in self-directed and independent ways for mental health support. Although users’ perspectives on CMHBs have been extensively examined and systematically synthesized, relatively little research has focused on how health care professionals (HCPs) perceive these tools. To develop a more comprehensive understanding of the implications of using CMHBs, the perspectives of HCPs should also be considered. As HCPs’ views are informed by their clinical expertise and professional responsibility, they may point to underexplored implications related to the safety, ethical use, and implementation of these tools.

**Objective:**

This paper presents the protocol for a systematic review that aims to identify, synthesize, and critically appraise the available evidence on HCPs’ perspectives regarding the use of CMHBs for mental health support, including perceptions of their therapeutic role, trustworthiness, safety, risks and benefits, ethical concerns, and implementation barriers and facilitators. Where relevant, the completed review will discuss its findings in relation to the existing literature on user perspectives to contextualize possible areas of convergence and divergence.

**Methods:**

A systematic review of the literature will be conducted in accordance with the PRISMA (Preferred Reporting Items for Systematic Reviews and Meta-Analyses) 2020 guidelines. Peer-reviewed qualitative, quantitative, and mixed methods studies will be identified through searches of PubMed (MEDLINE), PsycInfo, Embase, CINAHL, Web of Science, and Scopus, with no restrictions on publication date. Study screening will be supported by AI-assisted active learning using ASReview, following the SAFE stopping procedure, with independent quality-assurance (QA) screening by a second reviewer. Data will be synthesized using a convergent integrated mixed methods approach, and the findings will be reported narratively. Methodological quality will be appraised using the Mixed Methods Appraisal Tool (MMAT; version 2018).

**Results:**

The search for this review was conducted and completed in late November 2025 and identified 24,905 records before deduplication and 18,535 records after deduplication. The initial title and abstract screening began in January 2026 and is ongoing. Data extraction is expected to be completed by August 2026.

**Conclusions:**

This protocol outlines a systematic review that will synthesize the available empirical evidence on HCPs’ perspectives on the use of CMHBs for mental health support. The completed review aims to identify aspects such as perceived benefits, barriers and facilitators, and ethical concerns. By integrating qualitative, quantitative, and mixed methods evidence, the review will contribute to a more comprehensive understanding of the implementation and broader implications of CMHBs in mental health care.

## Introduction

### Background

Globally, mental health disorders (MHDs) are increasing and have become a major public health concern. Currently, more than 1 billion people are living with an MHD (World Health Organization [WHO]; [1]). Although MHDs can affect individuals across all ages and backgrounds, young adults and adolescents are disproportionately affected [2], with depression and anxiety accounting for the greatest burden [1,2]. Since 2010, the prevalence of anxiety disorders among adolescents aged 15 to 19 years has increased by approximately 70%, while rates of depression have risen by approximately 30% [3].

MHDs are associated with a wide range of adverse outcomes, including substance misuse [4], reduced quality of life [5,6], increased risk of physical illness [7,8], and increased suicide risk [9-11]. Beyond the individual level, the societal impact of MHDs is substantial. The economic burden is driven largely by health care expenditure and, to an even greater extent, by productivity losses attributable to common MHDs such as anxiety and depression [12,13]. These costs are estimated to amount to approximately US $1 trillion annually in lost productivity [1].

Professional mental health support, such as psychotherapy, is essential for the prevention and treatment of MHDs. However, despite the high and rapidly increasing burden of MHDs worldwide, many affected individuals who could benefit from such support do not receive adequate or effective care [14-17]. There are several barriers to receiving professional mental health support. Socioeconomic factors represent a major obstacle, as treatment costs, limited insurance coverage, and expensive copayment plans may make it impossible for some individuals to access care [18]. Stigma related to mental health conditions may also deter individuals from seeking professional support due to concerns about judgment or social consequences [19,20]. Other challenges include structural barriers such as shortages of mental health care providers [21], long wait times [22], and limited availability of local services, as well as logistical barriers, including transportation difficulties [23,24] and time constraints that may make travel to treatment locations infeasible for some individuals [25].

Conversational mental health chatbots (CMHBs) are increasingly being regarded as promising tools for addressing some of these barriers. Recent advances in AI, particularly in large language models (LLMs), have contributed to the development of these increasingly sophisticated chatbots capable of engaging in naturalistic conversational interactions [26,27]. Apps such as Wysa, Woebot, and Tess are aimed at providing on-demand, AI-powered mental health support and may deliver interventions informed by evidence-based approaches, such as cognitive behavioral therapy (CBT) or dialectical behavior therapy (DBT).

Many studies have examined users’ perspectives on CMHBs, with several systematic reviews synthesizing this user-centered body of research. CMHBs are often experienced as convenient [28,29], always available [30,31], and easier to approach than traditional mental health services. This is partly because they can feel more private and less stigmatizing when discussing sensitive concerns. Users commonly value this “judgment-free” space and the ability to engage in self-disclosure on demand [32,33]. In addition, users report appreciating the immediate emotional validation these tools can provide, as well as the possibility of receiving practical coping strategies [32].

At the same time, users also express several concerns. These include data privacy and confidentiality, particularly regarding how sensitive mental health data are stored and used [31,34]; mismatches between user expectations and the type or depth of support provided by CMHBs [35]; and a perceived lack of meaningful personalization, especially when users expect the chatbot to grasp nuance yet experience its responses as overly generic or scripted [28,33].

However, user perspectives by themselves may not fully capture the clinical and ethical risks associated with using these tools for mental health support. As users generally lack clinical knowledge or psychological expertise, they may be less likely to adequately perceive certain risks and limitations connected to using these tools, such as the misjudgment of symptom severity [36], inappropriate guidance [37], or overreliance on CMHBs in ways that could delay or substitute for professional care in cases that would require human clinical intervention [38,39].

To date, relatively little systematic attention has been paid to the perspectives of health care professionals (HCPs) regarding the use of CMHBs for mental health support. Yet understanding HCPs’ attitudes, opinions, and perceptions is essential for identifying additional potential risks, benefits, ethical concerns, and implementation barriers associated with the use of CMHBs. Their perspectives can contribute to a more comprehensive understanding of the implications of these tools.

### Objectives

This review aims to identify, synthesize, and critically appraise the available empirical evidence on the perspectives of HCPs regarding the use of CMHBs for mental health support, including perceptions of their therapeutic role, trustworthiness, safety, risks and benefits, ethical concerns, and implementation barriers and facilitators. Where relevant, the completed review will discuss its findings in relation to the existing literature on user perspectives to contextualize possible areas of convergence and divergence.

## Methods

### Overview

This systematic review will be reported in adherence to the PRISMA (Preferred Reporting Items for Systematic Reviews and Meta-Analyses) 2020 statement [40]. The protocol was developed using the PRISMA-P (Preferred Reporting Items for Systematic Review and Meta-Analysis Protocols) standard and is registered with PROSPERO (CRD420251180957) [41]. Any important protocol amendments will be documented, dated, and reported in PROSPERO and in the final publication.

### Eligibility Criteria

To define eligible studies, the population, intervention, comparator, outcome, and study design (PICOS) framework, as specified in the PRISMA (2020) statement, will be used. Detailed inclusion and exclusion criteria are presented in Table 1. Studies that do not fulfill the inclusion criteria will be removed from the review.

**Table 1.** Inclusion and exclusion criteria based on the population, intervention, comparator, outcome, and study design (PICOS) framework.

PICOS elements	Inclusion criteria	Exclusion criteria
Population	Studies involving HCPs^a^ who are qualified, licensed, registered, or undergoing supervised professional training Mixed-stakeholder studies if HCP data are reported separately or can be clearly extracted	Studies involving only patients; general users; students outside relevant professional training; developers; researchers; or technical experts without a clinical, therapeutic, or care-delivery role Studies in which HCP perspectives cannot be separated from those of other stakeholder groups
Intervention	CMHBs^b^, defined as interactive AI- or rule-based systems designed to provide mental health support Systems delivering structured or semistructured psychological techniques such as cognitive behavioral therapy, dialectical behavior therapy, mindfulness, meditation, or supportive dialogue	Chatbots unrelated to mental health Nonconversational digital systems such as symptom checkers, appointment schedulers, static psychoeducation apps, or purely informational systems General-purpose large language models, such as ChatGPT or Claude, unless incorporated into or adapted for use as purpose-built mental health interventions Tools used solely for diagnosis, screening, administration, or data collection without a conversational support component
Comparator	Studies with or without comparison groups if they address HCP perspectives on CMHBs Comparisons, where present, including human-delivered care, other digital mental health tools, or different chatbot types	Not applicable
Outcome	Studies reporting HCPs’ perspectives on CMHBs, including attitudes, opinions, perceptions, acceptability, trust, perceived concerns, and perceived barriers or facilitators to implementation	Studies reporting only technical performance, design or development processes, user engagement, use metrics, usability, diagnostic accuracy, or clinical or health outcomes without extractable HCP perspective data
Study design	Peer-reviewed primary empirical studies using qualitative, quantitative, or mixed methods designs Studies published in English, with no restrictions on publication date Systematic reviews conducted by others used only as a source for narrative references	Normative or theoretical articles; individual case studies; and non–peer-reviewed literature such as gray literature, blog posts, and newspapers

a HCP: health care professional.

b CMHB: conversational mental health chatbot.

### Search Strategy

A preliminary search strategy has been drafted by the research team and subsequently refined in consultation with an information specialist.

During the conceptualization phase of the review, 9 candidate seed studies were identified through preliminary scoping searches. These studies were discussed in team meetings to assess their relevance to the review aim and to refine the conceptual scope of the review. The seed studies were then used to inform the development of the search strategy, refine the search terms, and support the definition of the eligibility criteria before formal title and abstract screening. The final search strategy was subsequently tested against these seed studies to assess whether it retrieved known relevant records.

On the basis of consultation with the information specialist, the final search strategy was structured into three primary conceptual search blocks, closely aligned with the PICOS framework, to ensure that the search was sufficiently broad to capture all relevant literature while remaining focused enough to maintain relevance: (1) population, as defined in the eligibility criteria; (2) intervention, focusing on the technology being used and aiming to capture CMHBs as specified in the eligibility criteria; and (3) context, aiming to capture mental health and psychological aspects through general mental health search terms. This review is interested in all MHDs and specifically in the 5 most prevalent MHDs: anxiety disorders, depressive disorders, bipolar disorder, posttraumatic stress disorder (PTSD), and schizophrenia.

The following 6 databases were searched: PubMed (MEDLINE), PsycInfo, Embase, CINAHL, Web of Science, and Scopus. The 3 conceptual search blocks outlined previously were combined using the Boolean operator “AND.” The search used a combination of natural language vocabulary (keywords) and controlled vocabulary terms (subject headings), where available. Controlled vocabulary terms, such as MeSH terms, were adapted to the indexing systems of each database; when no equivalent subject headings existed, these terms were excluded. Searches were conducted using titles and abstracts. The full search strategy, including database-specific adaptations, is provided in Multimedia Appendix 1. A draft of the search strategy used for PubMed (MEDLINE) is presented in Textbox 1.

Textbox 1. **Search strategy used for PubMed (MEDLINE).**

**Population**

(“Health Personnel”[Mesh] OR “Social Workers”[Mesh] OR “healthcare professional*”[tiab] OR “health care professional*”[tiab] OR “health professional*”[tiab] OR clinician*[tiab] OR “healthcare worker*”[tiab] OR “health care worker*”[tiab] OR “healthcare staff”[tiab] OR “health care staff”[tiab] OR “medical staff”[tiab] OR doctor*[tiab] OR “nursing staff*”[tiab] OR “clinical staff”[tiab] OR provider*[tiab] OR “health care provider*”[tiab] OR “healthcare provider*”[tiab] OR physician*[tiab] OR “general practitioner*”[tiab] OR psychiatrist*[tiab] OR psychologist*[tiab] OR psychotherapist*[tiab] OR “mental health professional*”[tiab] OR “mental health practitioner*”[tiab] OR “medical practitioner*”[tiab] OR “behavioral health professional*”[tiab] OR “behavioural health professional*”[tiab] OR “medical professional*”[tiab] OR therapist*[tiab] OR counsellor*[tiab] OR counselor*[tiab] OR “social worker*”[tiab] OR nurse*[tiab] OR “nursing staff*”[tiab] OR “allied health professional*”[tiab] OR “allied health personnel”[tiab])

**Intervention**

(“Artificial Intelligence”[Mesh] OR chatbot*[tiab] OR “chat bot*”[tiab] OR “Chat bots”[tiab] OR “Chat-bot*”[tiab] OR chatterbot*[tiab] OR “chatter bot”[tiab] OR “chatter bots”[tiab] OR “large language model*”[tiab] OR gpt[tiab] OR chatgpt*[tiab] OR “conversational agent*”[tiab] OR “conversational bot”[tiab] OR “conversational bots”[tiab] OR “conversational system*”[tiab] OR “conversational interface*”[tiab] OR “conversational AI”[tiab] OR “conversational artificial intelligence”[tiab] OR “embodied conversational agent*”[tiab] OR “smartbot*”[tiab] OR “smart bot”[tiab] OR “smart bots”[tiab] OR “smart-bot*”[tiab] OR “virtual coach*”[tiab] OR “virtual agent*”[tiab] OR “virtual human*”[tiab] OR “virtual character*”[tiab] OR “virtual counsel*”[tiab] OR “embodied agent*”[tiab] OR “relational agent*”[tiab] OR “AI companion*”[tiab] OR “AI counsel*”[tiab] OR “dialogue system*”[tiab] OR “dialog system*”[tiab] OR Tess*[tiab] OR Wysa*[tiab] OR Woebot*[tiab] OR Therabot*[tiab] OR Youper*[tiab] OR vivibot*[tiab] OR Replika*[tiab] OR x2ai[tiab])

**Context**

(“Mental Health”[Mesh] OR “Mental Disorders”[Mesh] OR “mental health*”[tiab] OR “mental illness*”[tiab] OR “mental disorder*”[tiab] OR “mental well*”[tiab] OR wellbeing*[tiab] OR “well-being*”[tiab] OR psycholog*[tiab] OR therap*[tiab] OR emotion*[tiab] OR anxi*[tiab] OR “panic disorder*”[tiab] OR depress*[tiab] OR bipolar[tiab] OR “manic depress*”[tiab] OR mania[tiab] OR PTSD[tiab] OR “posttraumatic stress”[tiab] OR “post-traumatic stress”[tiab] OR “post traumatic stress”[tiab] OR schizophr*[tiab] OR psychosis[tiab] OR psychotic[tiab])

### Study Selection

All literature search results were uploaded to Covidence (Veritas Health Innovation), a web-based platform that supports the organization and management of systematic reviews, to remove duplicate records. The deduplicated dataset was then exported from Covidence and imported into ASReview. ASReview is an open-source machine learning tool designed to optimize title and abstract screening of the literature. Using a human-in-the-loop approach, it combines reviewer judgments with model-based prioritization. Through active learning, the tool continuously updates its predictions based on reviewer labels and presents the studies most likely to be relevant for manual evaluation [42]. The tool learns from the input of the reviewer as well as the protocol’s inclusion and exclusion criteria. It seeks to provide reviewers with the next potentially relevant article first.

To reduce the risk of missing relevant studies, the criteria for ending title and abstract screening will be guided by the SAFE procedure proposed by Boetje and van de Schoot [43]. This is a conservative set of stopping heuristics that offers a clear guideline for determining when to end the active-learning process in screening software such as ASReview [43]. Screening and the subsequent cessation of the screening process will proceed in four phases according to SAFE:

1. Random warm-up sampling: after deduplication in Covidence, a random sample of approximately 1% of all records will be screened manually to generate labeled training data for ASReview and to estimate the expected proportion of relevant records. If the random sample does not contain at least 1 relevant and 1 irrelevant record, additional randomly selected records (approximately 1%) will be screened until both classes are represented. This labeled warm-up sample will then be imported into ASReview as training data for the active-learning process.
2. Primary active-learning screening: active-learning screening in ASReview will continue until all of the following criteria are met: (1) all predefined seed studies have been retrieved, (2) at least twice the estimated number of relevant records have been screened, (3) at least 10% of the full dataset has been screened, and (4) no additional relevant studies have been identified in ≥50 consecutive screened records.
3. Model-switch safety pass: the remaining unscreened records will be reranked with a different AI model. Screening will continue until no relevant studies have been identified in ≥50 consecutive screened records.
4. Quality-control pass: all previously excluded records will be reranked using a third AI model. The highest-ranked subset will be manually reviewed until no additional relevant records have been identified in ≥50 consecutive screened records.

The SAFE procedure recommends using a computationally efficient and cheaper model for the primary active-learning screening phase (phase 2), followed by a computationally more demanding semantic model for the model-switch safety pass (phase 3) [43]. Accordingly, the screening in phase 2 will be conducted using ASReview’s default ELAS u4 (Electronic Learning Assistant Ultra version 3) model, consisting of the following settings: term frequency–inverse document frequency (TF-IDF) with bigrams as the feature extractor, a support vector machine as the classifier, Maximum as the query strategy, and Balanced as the balancing strategy.

In phase 3, the model-switch safety pass will be conducted using the model ELAS h3 (Electronic Learning Assistant Heavy version 3), which is a heavier semantic model, consisting of the following settings: mxbai-embed-large-v1 as the feature extractor, a support vector machine as the classifier, Maximum as the query strategy, and Balanced as the balancing strategy. This model requires the “Dory” extension of ASReview and is computationally more demanding.

In phase 4 (quality-control pass), records previously labeled as excluded will be reranked using the model ELAS u3 (Electronic Learning Assistant Ultra version 3), consisting of the following settings: TF-IDF as the feature extractor, a naive Bayes classifier, Maximum as the query strategy, and Balanced as the balancing strategy. ELAS u3 will be selected here because it provides a simpler and algorithmically distinct reranking of previously excluded records, thereby offering an additional safeguard against false exclusions without relying on the same model used during the primary screening phase.

The first reviewer will conduct the majority of screening within ASReview following the SAFE procedure. ASReview will be used only to prioritize the order in which records are presented for manual title and abstract screening and will not make final inclusion or exclusion decisions. A second reviewer will contribute independent screening at two predefined quality-assurance (QA) checkpoints: (1) both reviewers will independently screen the SAFE phase 1 random warm-up sample, and (2) intercoder reliability will be assessed using Cohen kappa. Any discrepancies at this stage will be resolved before continuing to the active-learning screening (phase 2) of the SAFE procedure; where consensus cannot be reached, a third reviewer will adjudicate.

During active-learning screening (phase 2), all records labeled as included, unclear, or potentially eligible by the first reviewer will be independently checked by a second reviewer. Disagreements at this stage will be resolved by discussion and, where necessary, by third reviewer adjudication.

After completion of all 4 SAFE phases, a second reviewer will independently rescreen a random sample of 10% of records that were initially excluded by the first reviewer to assess the safety of the stopping decision. Disagreements at this stage will also be resolved through discussion and, if necessary, adjudication by a third reviewer.

If the QA sample identifies records judged eligible or potentially eligible after consensus or third-reviewer adjudication, then these cases will be treated as possible false exclusions, and the stopping decision will be reassessed. Depending on the number and nature of such discrepancies, additional excluded records will then be reranked, and a further subset will be screened. Screening will continue until no further eligible or potentially eligible records are identified over the predefined consecutive-record stopping interval. All discrepancies, adjudication outcomes, clarifications or reassessments of the screening rules, and any extension of screening will be documented and reported in the final review.

### Data Extraction

Data extraction will be conducted independently by 2 reviewers using a pilot-tested, standardized data extraction form developed specifically for this review. Discrepancies will be resolved through discussion and, where necessary, adjudication by a third reviewer. The extraction form will capture metadata (eg, author, year, and publication details); methodological features; intervention characteristics (eg, chatbot type, function, LLM-based or rule-based, and the intended therapeutic role of the tool); study design; setting; HCP group or role; population details; sample size; and findings relevant to the review question.

For quantitative studies, extracted data will include measures used to assess HCPs’ perspectives (eg, acceptability, trust, and perceived risks and benefits); key numerical results (eg, summary statistics and effectiveness estimates); as well as information relevant to study quality or risk of bias.

For qualitative studies, extracted data will include author-reported themes; conceptual frameworks; categories; analytic approach; and illustrative findings. For mixed methods studies, both quantitative and qualitative components will be extracted separately before integration during synthesis.

Because HCPs are not a homogeneous group, professional background and level of training will be extracted wherever reported. Where studies provide disaggregated findings, these differences will be reported separately. Where studies report only aggregate HCP perspectives, the sample composition will be described, and the lack of profession-specific data will be considered when interpreting the findings. Formal subgroup analyses will only be undertaken if the included studies provide sufficiently comparable data.

Any amendments will be documented. Unclear or missing data will be recorded. Extracted data will be managed using spreadsheet software such as Excel (version 2408; Microsoft Inc), as well as qualitative data analysis software such as MAXQDA (version 26.2.1; VERBI Software GmbH), to support transparent synthesis.

### Data Analysis and Synthesis

Findings will be synthesized using a convergent integrated mixed methods approach, as outlined in the Joanna Briggs Institute (JBI) Manual for Evidence Synthesis [44]. This approach was selected because the review is expected to include qualitative, quantitative, and mixed methods studies reporting HCPs’ perspectives on CMHBs.

Quantitative findings will first be summarized descriptively in tables, using the statistics reported in the included studies where available. These findings will then be qualitized where necessary and appropriate. This means that numerical findings will be transformed into textual statements while preserving the direction and context of the original result. Where qualitizing would obscure important measurement details, numerical findings will be retained in summary tables and discussed narratively.

Qualitative findings and qualitized quantitative findings will be integrated using thematic synthesis [45]. Coding will combine deductive and inductive approaches. Deductive coding will be informed by predefined themes relevant to the review question, including perceptions of the therapeutic role of the CMHBs, trust in CMHBs, perceived safety, risks and benefits, ethical concerns, and implementation barriers and facilitators. Inductive coding will allow additional themes to be generated directly from the included studies, which involves the following steps: familiarization with the extracted data, generation of initial codes, grouping into descriptive themes, reviewing themes, and development of higher-order analytical themes.

The integrated findings will be organized in a synthesis matrix structured around shared outcome domains (eg, perceptions of the therapeutic role of CMHBs, trust in CMHBs, perceived safety, risks and benefits, ethical concerns, and implementation barriers and facilitators). The matrix will be used to map qualitative findings and qualitized quantitative findings across study characteristics (eg, professional group, clinical setting, chatbot type, and intended use of the chatbot).

Narrative synthesis will be used to present and interpret the integrated findings, supported by the summary tables and synthesis matrix [46]. Meta-analysis is not anticipated because the review is expected to include heterogeneous study designs, professional groups, chatbot interventions, and outcome measures. Meta-analysis will only be considered if at least 3 studies report sufficiently comparable quantitative outcomes using similar populations, measures, chatbot interventions, and study designs. If meta-analysis is not appropriate, quantitative findings will be, as mentioned before, summarized in tables and integrated narratively with the qualitative findings.

Conflicting findings will not be resolved by automatically prioritizing 1 type of evidence over another. Rather, discrepancies will be examined in relation to, for example, study design, professional group, clinical setting, chatbot type and use, outcome measurement, and methodological quality. Divergent findings will be reported transparently and discussed as part of the interpretation of the evidence.

Existing systematic reviews on user perspectives of CMHBs will not be included as primary data sources in the synthesis unless they meet the eligibility criteria for this review. However, where relevant, they may be used in the Discussion section to contextualize the findings and to identify possible areas of convergence or divergence between HCP and user perspectives. This comparison will be narrative and interpretive rather than constituting a formal comparative synthesis.

### Assessment of Methodological Quality

#### Risk of Bias Assessment

The extracted data will be critically appraised for methodological quality using the Mixed Methods Appraisal Tool (MMAT), version 2018 [47]. The MMAT was selected because the review will include qualitative, quantitative, and mixed methods primary studies. Each study will first be classified according to the applicable MMAT study category. Two reviewers will independently conduct the appraisal for each included study. Disagreements will be resolved through discussion and, where necessary, adjudication by a third reviewer.

#### Reporting Bias Assessment

Formal statistical assessment of publication bias will not be conducted because the review is expected to include heterogeneous quantitative, qualitative, and mixed methods evidence that will be integrated thematically.

#### Certainty Assessment

Because the review will use a convergent integrated approach in which quantitative findings may be qualitized and integrated with qualitative findings, no formal GRADE (Grading of Recommendations Assessment, Development and Evaluation) or GRADE-CERQual (Confidence in the Evidence from Reviews of Qualitative Research) rating will be assigned to the review findings. The methodological limitations, coherence, relevance, and contradictory evidence supporting the principal findings will instead be considered narratively.

## Results

**Figure 1.**
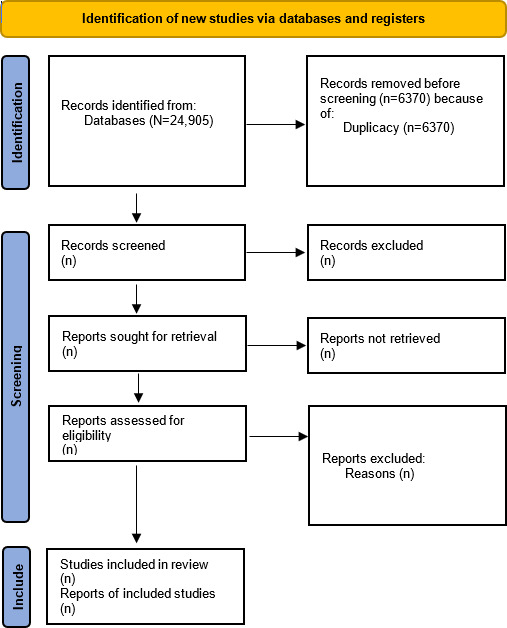
PRISMA (Preferred Reporting Items for Systematic Reviews and Meta-Analyses) flowchart.

This is a study protocol for a systematic review. Therefore, no results are available at the time of publication. The protocol was registered in PROSPERO in November 2025, before the database search. The search for this review was conducted and completed in late November 2025 and identified 24,905 records before deduplication and 18,535 records after deduplication. Minor adjustments were made to the protocol in December 2025; these did not affect study selection. The initial title and abstract screening began in January 2026 and is ongoing. Data extraction is expected to be completed by August 2026. The final results are expected to be published by November 2026. The identified studies will be reported in the PRISMA flowchart (Figure 1).

This work is supported as part of the National Centre of Competence in Research on Dependable, Ubiquitous Automation (NCCR Automation), funded by the Swiss National Science Foundation (grant 51NF40_225155).

## Discussion

### Anticipated Principal Findings

This protocol describes a systematic review that will synthesize empirical evidence on HCPs’ perspectives regarding the use of CMHBs as tools for mental health support. As the review has not yet been completed, no conclusions about the findings can be drawn at the stage of submission of this protocol.

However, on the basis of the preliminary literature informing this protocol, we anticipate that the included studies may identify a range of perceived benefits, risks, ethical concerns, and implementation-related considerations associated with the use of CMHBs. Potential areas of relevance may include HCPs’ views on the appropriate therapeutic role of these tools, their perceived usefulness, concerns about safety, trust in chatbot-generated responses, implications for the therapeutic relationship, and barriers and facilitators to implementation in mental health care.

### Comparison With Prior Work

Existing research on CMHBs has largely focused on users’ perspectives regarding, for example, experience or engagement [48]. Although user perspectives are essential for understanding uptake and acceptability, HCPs may evaluate these tools through a different lens, as their perspectives are additionally informed by their expertise and clinical responsibility. Therefore, this review will complement prior work by focusing specifically on this population group.

Where relevant, findings from this review will be discussed in relation to existing systematic reviews on user perspectives of CMHBs. These sources will not be included as primary data in the synthesis unless they meet the eligibility criteria, but they may be used to contextualize areas of convergence and divergence between HCP and user perspectives. Specifically, this comparison may, for example, help clarify whether HCPs and users emphasize similar concerns or whether HCPs place greater emphasis on issues such as clinical safety, accountability, and risk management.

### Strengths and Limitations

This review will address an important gap by focusing specifically on HCPs’ perspectives on the use of CMHBs, a topic that has received less systematic attention than user perspectives. This focus is crucial, as HCPs may evaluate CMHBs considerably differently and thus, may point to underexplored implications of using such tools. This may help inform future research and guide discussions around the safe, ethical, and responsible integration of CMHBs into mental health care.

Including qualitative, quantitative, and mixed methods studies will allow a broad synthesis of evidence across different methodological approaches. The use of the SAFE procedure as a conservative stopping heuristic for title and abstract screening in ASReview may help reduce the risk of prematurely stopping the screening process and missing relevant studies. Any decisions related to screening, data extraction, synthesis, and deviations from the protocol will be documented and reported transparently in the completed review.

On the other hand, several limitations should also be acknowledged. The review will only include peer-reviewed studies published in English, which may introduce publication and language bias and may exclude other relevant literature. Another limitation concerns the use of ASReview. Although a conservative stopping heuristic and safeguards, including independent QA screening by a second reviewer, will be used to reduce the risk of missing relevant studies, it should be noted that this approach is not fully equivalent to dual independent title and abstract screening of all records. This limitation will be considered when interpreting the comprehensiveness and reproducibility of the screening process.

Finally, heterogeneity across, for example, professional groups, clinical settings, chatbot types, and study designs is expected. This may limit comparability across studies and is likely to restrict the possibility of meta-analysis.

## Supplementary material

10.2196/92780 Multimedia Appendix 1 Search strategies for each database.

10.2196/92780 Checklist 1 PRISMA-P 2015 checklist.

## Abbreviations

CBT: cognitive behavioral therapy
CERQual: Confidence in the Evidence from Reviews of Qualitative Research
CMHB: conversational mental health chatbot
DBT: dialectical behavior therapy
ELAS h3: Electronic Learning Assistant Heavy version 3
ELAS u3: Electronic Learning Assistant Ultra version 3
ELAS u4: Electronic Learning Assistant Ultra version 4
GRADE: Grading of Recommendations Assessment, Development and Evaluation
HCP: health care professional
JBI: Joanna Briggs Institute
LLM: large language model
MHD: mental health disorder
MMAT: Mixed Methods Appraisal Tool
NCCR Automation: National Centre of Competence in Research on Dependable, Ubiquitous Automation
PICOS: population, intervention, comparator, outcome, and study design
PRISMA: Preferred Reporting Items for Systematic Reviews and Meta-Analyses
PRISMA-P: Preferred Reporting Items for Systematic Review and Meta-Analysis Protocols
PTSD: posttraumatic stress disorder
QA: quality-assurance
TF-IDF: term frequency–inverse document frequency
WHO: World Health Organization
