# Supplementary material for: Health Care Professionals’ Perspectives on Conversational Mental Health Chatbots: Protocol for a Systematic Review
Source: JMIR Res Protoc. 2026 Aug 14;15:e92780. doi: 10.2196/92780 (PMC13475783; doi:10.2196/92780)
Supplement: Supplementary file 1 [file resprot-v15-e92780-s001.docx]

# Additional file 2

## Search Strategies for Each Database

| **Database** | **Population** | **Intervention** | **Context** |
| --- | --- | --- | --- |
| **Pubmed/Medline** | ("Health Personnel"[Mesh] OR "Social Workers"[Mesh] OR "healthcare professional*"[tiab] OR "health care professional*"[tiab] OR "health professional*"[tiab] OR clinician*[tiab] OR "healthcare worker*"[tiab] OR "health care worker*"[tiab] OR "healthcare staff"[tiab] OR "health care staff"[tiab] OR "medical staff"[tiab] OR doctor*[tiab] OR "nursing staff*"[tiab] OR "clinical staff"[tiab] OR provider*[tiab] OR "health care provider*"[tiab] OR "healthcare provider*"[tiab] OR physician*[tiab] OR "general practitioner*"[tiab] OR psychiatrist*[tiab] OR psychologist*[tiab] OR psychotherapist*[tiab] OR "mental health professional*"[tiab] OR "mental health practitioner*"[tiab] OR "medical practitioner*"[tiab] OR "behavioral health professional*"[tiab] OR "behavioural health professional*"[tiab] OR "medical professional*"[tiab] OR therapist*[tiab] OR counsellor*[tiab] OR counselor*[tiab] OR "social worker*"[tiab] OR nurse*[tiab] OR "nursing staff*"[tiab] OR "allied health professional*"[tiab] OR "allied health personnel"[tiab]) | ("Artificial Intelligence"[Mesh] OR chatbot*[tiab] OR "chat bot*"[tiab] OR "Chat bots"[tiab] OR "Chat-bot*"[tiab] OR chatterbot*[tiab] OR "chatter bot"[tiab] OR "chatter bots"[tiab] OR "large language model*"[tiab] OR gpt[tiab] OR chatgpt*[tiab] OR "conversational agent*"[tiab] OR "conversational bot"[tiab] OR "conversational bots"[tiab] OR "conversational system*"[tiab] OR "conversational interface*"[tiab] OR "conversational AI"[tiab] OR "conversational artificial intelligence"[tiab] OR "embodied conversational agent*"[tiab] OR "smartbot*"[tiab] OR "smart bot"[tiab] OR "smart bots"[tiab] OR "smart-bot*"[tiab] OR "virtual coach*"[tiab] OR "virtual agent*"[tiab] OR "virtual human*"[tiab] OR "virtual character*"[tiab] OR "virtual counsel*"[tiab] OR "embodied agent*"[tiab] OR "relational agent*"[tiab] OR "AI companion*"[tiab] OR "AI counsel*"[tiab] OR "dialogue system*"[tiab] OR "dialog system*"[tiab] OR Tess*[tiab] OR Wysa*[tiab] OR Woebot*[tiab] OR Therabot*[tiab] OR Youper*[tiab] OR vivibot*[tiab] OR Replika*[tiab] OR x2ai[tiab]) | ("Mental Health"[Mesh] OR "Mental Disorders"[Mesh] OR "mental health*"[tiab] OR "mental illness*"[tiab] OR "mental disorder*"[tiab] OR "mental well*"[tiab] OR wellbeing*[tiab] OR "well-being*"[tiab] OR psycholog*[tiab] OR therap*[tiab] OR emotion*[tiab] OR anxi*[tiab] OR "panic disorder*"[tiab] OR depress*[tiab] OR bipolar[tiab] OR "manic depress*"[tiab] OR mania[tiab] OR PTSD[tiab] OR "posttraumatic stress"[tiab] OR "post-traumatic stress"[tiab] OR "post traumatic stress"[tiab] OR schizophr*[tiab] OR psychosis[tiab] OR psychotic[tiab]) |
| **PsycINFO (Ovid)** | (exp Health Personnel/ or exp Social Workers/ or (healthcare professional* or health care professional* or health professional* or clinician* or healthcare worker* or health care worker* or healthcare staff or health care staff or medical staff or doctor* or nursing staff* or clinical staff or provider* or health care provider* or healthcare provider* or physician* or general practitioner* or psychiatrist* or psychologist* or psychotherapist* or mental health professional* or mental health practitioner* or medical practitioner* or behavioral health professional* or behavioural health professional* or medical professional* or therapist* or counsellor* or counselor* or social worker* or nurse* or nursing staff* or allied health professional* or allied health personnel).ti,ab.) | (exp Artificial Intelligence/ or (chatbot* or chat bot* or chat bots or chat-bot* or chatterbot* or chatter bot or chatter bots or large language model* or gpt or chatgpt* or conversational agent* or conversational bot or conversational bots or conversational system* or conversational interface* or conversational AI or conversational artificial intelligence or embodied conversational agent* or smartbot* or smart bot or smart bots or smart-bot* or virtual coach* or virtual agent* or virtual human* or virtual character* or virtual counsel* or embodied agent* or relational agent* or AI companion* or AI counsel* or dialogue system* or dialog system* or Tess* or Wysa* or Woebot* or Therabot* or Youper* or vivibot* or Replika* or x2ai).ti,ab.) | (exp Mental Health/ or exp Mental Disorders/ or (mental health* or mental illness* or mental disorder* or mental well* or wellbeing* or well-being* or psycholog* or therap* or emotion* or anxi* or panic disorder* or depress* or bipolar or manic depress* or mania or PTSD or posttraumatic stress or post-traumatic stress or post traumatic stress or schizophr* or psychosis or psychotic).ti,ab.) |
| **Embase** | ('health personnel'/exp OR 'social worker'/exp OR ("healthcare professional*":ti,ab OR "health care professional*":ti,ab OR "health professional*":ti,ab OR clinician*:ti,ab OR "healthcare worker*":ti,ab OR "health care worker*":ti,ab OR "healthcare staff":ti,ab OR "health care staff":ti,ab OR "medical staff":ti,ab OR doctor*:ti,ab OR "nursing staff*":ti,ab OR "clinical staff":ti,ab OR provider*:ti,ab OR "health care provider*":ti,ab OR "healthcare provider*":ti,ab OR physician*:ti,ab OR "general practitioner*":ti,ab OR psychiatrist*:ti,ab OR psychologist*:ti,ab OR psychotherapist*:ti,ab OR "mental health professional*":ti,ab OR "mental health practitioner*":ti,ab OR "medical practitioner*":ti,ab OR "behavioral health professional*":ti,ab OR "behavioural health professional*":ti,ab OR "medical professional*":ti,ab OR therapist*:ti,ab OR counsellor*:ti,ab OR counselor*:ti,ab OR "social worker*":ti,ab OR nurse*:ti,ab OR "allied health professional*":ti,ab OR "allied health personnel":ti,ab)) | ('artificial intelligence'/exp OR (chatbot*:ti,ab OR "chat bot*":ti,ab OR "chat bots":ti,ab OR "chat-bot*":ti,ab OR chatterbot*:ti,ab OR "chatter bot":ti,ab OR "chatter bots":ti,ab OR "large language model*":ti,ab OR gpt:ti,ab OR chatgpt*:ti,ab OR "conversational agent*":ti,ab OR "conversational bot":ti,ab OR "conversational bots":ti,ab OR "conversational system*":ti,ab OR "conversational interface*":ti,ab OR "conversational ai":ti,ab OR "conversational artificial intelligence":ti,ab OR "embodied conversational agent*":ti,ab OR smartbot*:ti,ab OR "smart bot":ti,ab OR "smart bots":ti,ab OR "smart-bot*":ti,ab OR "virtual coach*":ti,ab OR "virtual agent*":ti,ab OR "virtual human*":ti,ab OR "virtual character*":ti,ab OR "virtual counsel*":ti,ab OR "embodied agent*":ti,ab OR "relational agent*":ti,ab OR "ai companion*":ti,ab OR "ai counsel*":ti,ab OR "dialogue system*":ti,ab OR "dialog system*":ti,ab OR tess*:ti,ab OR wysa*:ti,ab OR woebot*:ti,ab OR therabot*:ti,ab OR youper*:ti,ab OR vivibot*:ti,ab OR replika*:ti,ab OR x2ai:ti,ab)) | ('mental health'/exp OR 'mental disease'/exp OR ("mental health*":ti,ab OR "mental illness*":ti,ab OR "mental disorder*":ti,ab OR "mental well*":ti,ab OR wellbeing*:ti,ab OR "well-being*":ti,ab OR psycholog*:ti,ab OR therap*:ti,ab OR emotion*:ti,ab OR anxi*:ti,ab OR "panic disorder*":ti,ab OR depress*:ti,ab OR bipolar:ti,ab OR "manic depress*":ti,ab OR mania:ti,ab OR ptsd:ti,ab OR "posttraumatic stress":ti,ab OR "post-traumatic stress":ti,ab OR "post traumatic stress":ti,ab OR schizophr*:ti,ab OR psychosis:ti,ab OR psychotic:ti,ab)) |
| CINAHL | (MH "Health Personnel+" OR MH "Social Workers" OR MH "Health Occupations+" OR TI ("healthcare professional*" OR "health care professional*" OR "health professional*" OR clinician* OR "healthcare worker*" OR "health care worker*" OR "healthcare staff" OR "health care staff" OR "medical staff" OR doctor* OR "nursing staff*" OR "clinical staff" OR provider* OR "health care provider*" OR "healthcare provider*" OR physician* OR "general practitioner*" OR psychiatrist* OR psychologist* OR psychotherapist* OR "mental health professional*" OR "mental health practitioner*" OR "medical practitioner*" OR "behavioral health professional*" OR "behavioural health professional*" OR "medical professional*" OR therapist* OR counsellor* OR counselor* OR "social worker*" OR nurse* OR "nursing staff*" OR "allied health professional*" OR "allied health personnel") OR AB ("healthcare professional*" OR "health care professional*" OR "health professional*" OR clinician* OR "healthcare worker*" OR "health care worker*" OR "healthcare staff" OR "health care staff" OR "medical staff" OR doctor* OR "nursing staff*" OR "clinical staff" OR provider* OR "health care provider*" OR "healthcare provider*" OR physician* OR "general practitioner*" OR psychiatrist* OR psychologist* OR psychotherapist* OR "mental health professional*" OR "mental health practitioner*" OR "medical practitioner*" OR "behavioral health professional*" OR "behavioural health professional*" OR "medical professional*" OR therapist* OR counsellor* OR counselor* OR "social worker*" OR nurse* OR "nursing staff*" OR "allied health professional*" OR "allied health personnel")) | (MH "Artificial Intelligence+" OR MH "Natural Language Processing" OR MH "Decision Support Systems, Clinical" OR TI (chatbot* OR "chat bot*" OR "Chat bots" OR "Chat-bot*" OR chatterbot* OR "chatter bot" OR "chatter bots" OR "large language model*" OR gpt OR chatgpt* OR "conversational agent*" OR "conversational bot" OR "conversational bots" OR "conversational system*" OR "conversational interface*" OR "conversational AI" OR "conversational artificial intelligence" OR "embodied conversational agent*" OR smartbot* OR "smart bot" OR "smart bots" OR "smart-bot*" OR "virtual coach*" OR "virtual agent*" OR "virtual human*" OR "virtual character*" OR "virtual counsel*" OR "embodied agent*" OR "relational agent*" OR "AI companion*" OR "AI counsel*" OR "dialogue system*" OR "dialog system*" OR Tess* OR Wysa* OR Woebot* OR Therabot* OR Youper* OR vivibot* OR Replika* OR x2ai) OR AB (chatbot* OR "chat bot*" OR "Chat bots" OR "Chat-bot*" OR chatterbot* OR "chatter bot" OR "chatter bots" OR "large language model*" OR gpt OR chatgpt* OR "conversational agent*" OR "conversational bot" OR "conversational bots" OR "conversational system*" OR "conversational interface*" OR "conversational AI" OR "conversational artificial intelligence" OR "embodied conversational agent*" OR smartbot* OR "smart bot" OR "smart bots" OR "smart-bot*" OR "virtual coach*" OR "virtual agent*" OR "virtual human*" OR "virtual character*" OR "virtual counsel*" OR "embodied agent*" OR "relational agent*" OR "AI companion*" OR "AI counsel*" OR "dialogue system*" OR "dialog system*" OR Tess* OR Wysa* OR Woebot* OR Therabot* OR Youper* OR vivibot* OR Replika* OR x2ai)) | (MH "Mental Health+" OR MH "Mental Disorders+" OR MH "Psychiatric Nursing" OR MH "Psychotherapy" OR TI ("mental health*" OR "mental illness*" OR "mental disorder*" OR "mental well*" OR wellbeing* OR "well-being*" OR psycholog* OR therap* OR emotion* OR anxi* OR "panic disorder*" OR depress* OR bipolar OR "manic depress*" OR mania OR PTSD OR "posttraumatic stress" OR "post-traumatic stress" OR "post traumatic stress" OR schizophr* OR psychosis OR psychotic) OR AB ("mental health*" OR "mental illness*" OR "mental disorder*" OR "mental well*" OR wellbeing* OR "well-being*" OR psycholog* OR therap* OR emotion* OR anxi* OR "panic disorder*" OR depress* OR bipolar OR "manic depress*" OR mania OR PTSD OR "posttraumatic stress" OR "post-traumatic stress" OR "post traumatic stress" OR schizophr* OR psychosis OR psychotic)) |
| Web of Science | TS=(("healthcare professional*" OR "health care professional*" OR "health professional*" OR clinician* OR "healthcare worker*" OR "health care worker*" OR "healthcare staff" OR "health care staff" OR "medical staff" OR doctor* OR "nursing staff*" OR "clinical staff" OR provider* OR "health care provider*" OR "healthcare provider*" OR physician* OR "general practitioner*" OR psychiatrist* OR psychologist* OR psychotherapist* OR "mental health professional*" OR "mental health practitioner*" OR "medical practitioner*" OR "behavioral health professional*" OR "behavioural health professional*" OR "medical professional*" OR therapist* OR counsellor* OR counselor* OR "social worker*" OR nurse* OR "allied health professional*" OR "allied health personnel")) | TS=((chatbot* OR "chat bot*" OR "chat bots" OR "chat-bot*" OR chatterbot* OR "chatter bot" OR "chatter bots" OR "large language model*" OR gpt OR chatgpt* OR "conversational agent*" OR "conversational bot" OR "conversational bots" OR "conversational system*" OR "conversational interface*" OR "conversational ai" OR "conversational artificial intelligence" OR "embodied conversational agent*" OR smartbot* OR "smart bot" OR "smart bots" OR "smart-bot*" OR "virtual coach*" OR "virtual agent*" OR "virtual human*" OR "virtual character*" OR "virtual counsel*" OR "embodied agent*" OR "relational agent*" OR "ai companion*" OR "ai counsel*" OR "dialogue system*" OR "dialog system*" OR tess* OR wysa* OR woebot* OR therabot* OR youper* OR vivibot* OR replika* OR x2ai)) | TS=(("mental health*" OR "mental illness*" OR "mental disorder*" OR "mental well*" OR wellbeing* OR "well-being*" OR psycholog* OR therap* OR emotion* OR anxi* OR "panic disorder*" OR depress* OR bipolar OR "manic depress*" OR mania OR ptsd OR "posttraumatic stress" OR "post-traumatic stress" OR "post traumatic stress" OR schizophr* OR psychosis OR psychotic)) |
| **Scopus** | (TITLE-ABS-KEY("healthcare professional*" OR "health care professional*" OR "health professional*" OR clinician* OR "healthcare worker*" OR "health care worker*" OR "healthcare staff" OR "health care staff" OR "medical staff" OR doctor* OR "nursing staff*" OR "clinical staff" OR provider* OR "health care provider*" OR "healthcare provider*" OR physician* OR "general practitioner*" OR psychiatrist* OR psychologist* OR psychotherapist* OR "mental health professional*" OR "mental health practitioner*" OR "medical practitioner*" OR "behavioral health professional*" OR "behavioural health professional*" OR "medical professional*" OR therapist* OR counsellor* OR counselor* OR "social worker*" OR nurse* OR "allied health professional*" OR "allied health personnel")) | (TITLE-ABS-KEY(chatbot* OR "chat bot*" OR "chat bots" OR "chat-bot*" OR chatterbot* OR "chatter bot" OR "chatter bots" OR "large language model*" OR gpt OR chatgpt* OR "conversational agent*" OR "conversational bot" OR "conversational bots" OR "conversational system*" OR "conversational interface*" OR "conversational ai" OR "conversational artificial intelligence" OR "embodied conversational agent*" OR smartbot* OR "smart bot" OR "smart bots" OR "smart-bot*" OR "virtual coach*" OR "virtual agent*" OR "virtual human*" OR "virtual character*" OR "virtual counsel*" OR "embodied agent*" OR "relational agent*" OR "ai companion*" OR "ai counsel*" OR "dialogue system*" OR "dialog system*" OR tess* OR wysa* OR woebot* OR therabot* OR youper* OR vivibot* OR replika* OR x2ai)) | (TITLE-ABS-KEY("mental health*" OR "mental illness*" OR "mental disorder*" OR "mental well*" OR wellbeing* OR "well-being*" OR psycholog* OR therap* OR emotion* OR anxi* OR "panic disorder*" OR depress* OR bipolar OR "manic depress*" OR mania OR ptsd OR "posttraumatic stress" OR "post-traumatic stress" OR "post traumatic stress" OR schizophr* OR psychosis OR psychotic)) |

- Logic applied to all databases: **Population** AND **Intervention** AND **Context**
